# The Correlation Between Probiotics and Anxiety and Depression Levels in Cancer Patients: A Retrospective Cohort Study

**DOI:** 10.3389/fpsyt.2022.830081

**Published:** 2022-04-01

**Authors:** Ziqi Ye, Yanfang Zhang, Mengfei Du, Shaojia Lu, Qingwei Zhao, Si Yang

**Affiliations:** ^1^Department of Clinical Pharmacy, The First Affiliated Hospital, Zhejiang University School of Medicine, Hangzhou, China; ^2^Department of Psychiatry, The First Affiliated Hospital, Zhejiang University School of Medicine, Hangzhou, China; ^3^The Key Laboratory of Mental Disorder’s Management of Zhejiang Province, Zhejiang Engineering Center for Mathematical Mental Health, Hangzhou, China

**Keywords:** probiotics, anxiety, depression, cancer patients, gut microbiota

## Abstract

**Objective:**

Studies have shown a correlation between gut microbiota and anxiety and depression levels. However, these studies are mainly animal studies or clinical studies of non-cancer patients, there is still a lack of relevant studies in cancer patients. The main objective of this trial was to analyze the correlation between probiotics and anxiety and depression levels in cancer patients.

**Methods:**

We screened all cancer patients consecutively admitted to the inpatient department of the First Affiliated Hospital, Zhejiang University School of Medicine in May 2020. A total of 292 cancer patients met our inclusion criteria. Then, we followed up all patients for 24 weeks. Patients who had incomplete data or loss of follow-up were excluded. In addition, in patients who took probiotics, those did not take probiotics consistently or did not take specific probiotics were excluded. Ultimately, the number of patients enrolled was 82 in probiotics cohort and 100 in non-probiotics cohort. The 17-item Hamilton Depression Scale (HAMD-17) questionnaire was used to measure the depression levels of the patients, and we also used Hamilton Anxiety Scale (HAMA) questionnaire to assess the patients’ anxiety levels. A logistic regression model was used to analyze whether the difference in baseline data of two cohorts would affect the final result.

**Results:**

Demographic and clinical characteristics of all cancer patients enrolled in probiotics cohort and non-probiotics cohort were similar except the cancer therapy (*P* = 0.004). According to the HAMA score, we divided cancer patients into non-anxiety group (HAMA score < 14) and anxiety group (HAMA score ≥ 14). Similarly, cancer patients were also divided into non-depression group (HAMD-17 score ≤ 7) and depression group (HAMD-17 score > 7). The results demonstrated that there was no statistical difference in the proportion of patients with anxiety (6.1 and 13.0%, respectively, *P* = 0.121) and depression (30.5 and 23.0%, respectively, *P* = 0.254) between probiotics and non-probiotics cohorts. The results of logistic regression model analysis further proved that the baseline difference in cancer therapy did not affect the conclusions.

**Conclusion:**

Our results still suggest that there is no significant correlation between probiotics and anxiety and depression levels in cancer patients. Therefore, we do not recommend supplementing probiotics for cancer patients to prevent anxiety and depression. Moreover, high-quality RCTs are also needed to further confirm the conclusions of this study.

## Introduction

Cancer, a serious disease in which cells grow indefinitely, is one of the leading causes of death worldwide (1). Various factors affected the cancer patients such as unpredictability of tumor recurrence, stressful treatments and constant anticipatory threats (2–5). Therefore, emotional distress is a common symptom in cancer patients. Depression and anxiety are considered two common comorbidities of cancer, and could lead to many adverse events in the treatment of cancer, for example influencing life quality and negatively impacting medical adherence, cancer survival, and costs of treatments (6).

Several studies have reported the influence of the gastrointestinal microbiota on the gut-brain axis, and its possible function in neuropsychiatric disorders such as Alzheimer’s disease, anxiety, depression, autism, and Parkinson’s disease (7–10). It has been shown that compound probiotic bacteria could produce neurotransmitters and neuropeptides, including serotonin, Gamma-Amino Butyric Acid (GABA), and Brain-Derived Neurotrophic Factor (BDNF), which could regulate people’s emotion (11, 12). Several studies supported this theory that probiotic bacteria are effective on improving central nervous system (CNS) function, including functions associated with mental illness, such as anxiety, depression, memory capability, and stress response (13, 14).

However, the current studies on the correlation between probiotics and anxiety and depression are mostly animal studies, and human studies are still relatively lacking. In a previous study, healthy people were given a probiotics mixture, which included *B. longum R0175*, *Lactobacillus helveticus* R0052, or placebo for 30 days. Results revealed that the probiotics administration group showed significantly less emotional distress than control group (15). Similarly, in another clinical study, healthy subjects were treated randomly either a milk drink with probiotic or placebo for 3 weeks, and mood was assessed before treatment and after 10 and 20 days of administration. Subjects who initially presented with depression or anxiety showed significant improvement in mood disorders after receiving probiotics (16). However, there are still some disputes about whether probiotic can improve mood disorders. Therefore, meta-analysis was performed to get a comprehensive result. The results of a meta-analysis suggested that probiotics only benefit to animals, but do not significantly alleviate anxiety symptoms in healthy and anxious samples (17). Another meta-analysis also showed that probiotics only significantly improved depressive symptoms in patients with major depressive disorder, but not in patients with mild to moderate depression or in healthy people (18). In addition, another meta-analysis demonstrated that probiotics significantly improved symptoms in patients with mild to moderate depression, but not in healthy people (19).

The above studies suggested that the efficacy of probiotics may vary depending on the study population. Studies have shown that there are significant differences in the composition of the gut microbiome among cancer patients, psychiatric patients, and healthy individuals (20–22), which may be one of the main reasons for the differences in the efficacy of probiotic supplementation in these populations. Several studies explored the efficacy of probiotics for anxiety and depression in both psychiatric patients and healthy individuals, but there was still no literature on whether probiotics could play a preventive role in mood disorders of cancer patients. Therefore, the main objective of this study was to analyze the correlation between probiotics and anxiety and depression levels in cancer patients.

## Materials and Methods

### Participants

We screened all cancer patients consecutively admitted to the inpatient department of the First Affiliated Hospital, Zhejiang University School of Medicine in May 2020. The number of all cancer patients included was 292. A pathology report was required for each patient. The patients included in our study were admitted to our hospital but were then discharged. Then, we followed up all patients for 24 weeks whether they had been taking probiotics as prescribed and finally scored their anxiety and depression levels. The follow-up time points were the first, third and sixth months.

### Inclusion and Exclusion Criteria

The inclusion criteria for patients were: (1) 18–70 years old, no gender restrictions; (2) diagnosed with cancer; (3) no previous use of probiotics; (4) no previous diagnosis of anxiety or depression; (5) patients who were admitted to our hospital in May 2020. The exclusion criteria for all patients were: (1) incomplete medical records; (2) loss of follow-up; for patients who took probiotics were: (1) not taking probiotics consistently during follow-up; (2) not taking specific strains of *Lactobacillus* genera and *Bifidobacterium* genera. Patients who did not take probiotics during hospitalization and follow-up after discharge were included in the non-probiotics group.

### Probiotics

One study suggested that probiotics consisting of specific strains of *Lactobacillus* genera and *Bifidobacterium* genera might have the potential to prevent and treat depression and anxiety disorders (23). Therefore, probiotics in this study included compound eosinophil-lactobacillus tablets, and live combined bifidobacterium, lactobacillus and enterococcus capsules. These probiotics are commercial products, the types and dosages of probiotic strains are shown in Table 1. Patients took probiotics for 24 weeks according to the recommended dosage of drug instructions.

**TABLE 1:** The types and dosages of probiotic strains in our study.

Probiotics	Types and dosages of probiotic strains (1 tablet)	Company (Country)	Recommended dosage of drug instructions
(1) Compound eosinophil-lactobacillus tablets	(1) *Lactobacillus acidophilus*: 5.0 × 10^6^ CFU	Tonghua Golden-horse Pharmaceutical Group Co., Ltd. (China)	2 tablets, 3 times a day, take with warm water
(2) Live combined bifidobacterium, lactobacillus and enterococcus capsules	(1) *Bifidobacterium longum*: ≥ 1 × 10^7^ CFU (2) *Lactobacillus acidophilus*: ≥ 1 × 10^7^ CFU (3) *Enterococcus faecalis*: ≥ 1 × 10^7^ CFU	Shanghai Shangyao Xinyi Pharmaceutical Co., Ltd. (China)	3 tablets, twice a day, take with warm water half an hour after meals

*CFU, Colony-Forming Unit.*

### Measures

We reviewed the electronic medical records system and extracted socio-demographic information of eligible participants, including name, gender, age, smoking, drinking, education level, residence, cancer types, tumor size, tumor stage, and cancer therapy. Psychiatrist of our team assessed the depression and anxiety levels of these cancer patients by phone, and the 17-item Hamilton Depression Scale (HAMD-17) questionnaire was used to measure depression levels of the patients (24, 25). This questionnaire consists of 17 items, each with a score of 0–4. A total score less than 8 indicates no depression, 8–17 indicates mild depression, 18–24 indicates moderate depression, and more than 24 indicates severe depression. We also used Hamilton Anxiety Scale (HAMA) questionnaire to assess the patients’ anxiety levels (25). This questionnaire consists of 14 items, and each item is 0–4 points. When the total score less than 7 suggests no anxiety, the total score ≥ 7 suggests possible anxiety, the total score ≥ 14 suggests certain anxiety, the total score ≥ 21 suggests significant anxiety, and the total score ≥ 29 suggests severe anxiety.

### Statistical Analysis

The Kolmogorov–Smirnov test was performed to verify whether continuous variables conform to normal distribution. Mean ± Standard Deviation (SD) or median (interquartile range) was calculated for continuous variables, and frequency and percentage for categorical variables. We applied the Student’s *t*-test to compare the differences between probiotics cohort and non-probiotics cohort for continuous variables with normal distribution, otherwise, Mann-Whitney U test was used. Chi-square test or Fisher’s exact test was used for categorical variables. To assess the correlation between probiotics administration and anxiety and depression levels in cancer patients, we set the HAMD-17 score of 7 and the HAMA score of 14 as a cut-off value of depression and anxiety. Logistic regression models were used to analyze whether the difference in baseline data of two cohorts would affect the final result. The IBM Statistical Package for Social Sciences (SPSS, version 26) software was used to do all statistical analyses and two-tailed *p* < 0.05 was considered to be statistically significant.

## Results

### Demographic and Clinical Characteristics of All Cancer Patients Enrolled

**FIGURE 1:**
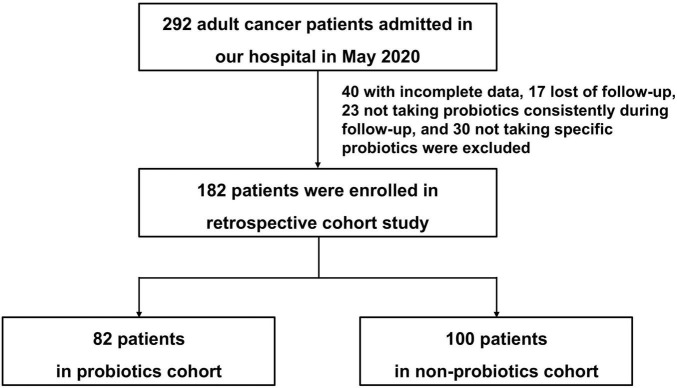
Flow chart of this study.

A total of 292 cancer patients at the First Affiliated Hospital, Zhejiang University School of Medicine in May 2020 met our inclusion criteria. We reviewed the electronic medical records system, 40 patients with incomplete data, 17 patients who lost follow-up in all patients were excluded. In addition, 23 patients who took probiotics irregularly during follow-up, and 30 patients who didn’t take the specific strains of *Lactobacillus* genera and *Bifidobacterium* genera in patients who took probiotics were excluded. Finally, the number of patients enrolled was 82 in probiotics cohort and 100 in non-probiotics cohort. The flow chart of this study was detailed in Figure 1.

Demographic and clinical characteristics of all cancer patients enrolled in probiotics and non-probiotics cohorts are shown in Table 2. The median age of patients in two cohorts was 64 and 63, respectively. Male patients (68.3 and 69.0% in probiotics cohort and non-probiotics cohort, respectively), patients with secondary education (53.8 and 57.3%), and patients living in city (63.4 and 71.0%) accounted for the majority of enrolled patients. The proportion of non-smokers (53.7 and 51.0% in probiotics cohort and non-probiotics cohort, respectively) and non-drinkers (69.5 and 70.0% in probiotics cohort and non-probiotics cohort, respectively) were relatively higher. The cancer types of the patients in these two cohorts mainly included liver cancer, pancreatic cancer, and lung cancer. There were no statistical differences in the demographic and clinical characteristics of the cancer patients enrolled in these two cohorts (*P* > 0.05) except the cancer therapy (*P* = 0.004).

**TABLE 2:** Demographic and clinical characteristics of all cancer patients enrolled.

Variables	Probiotics (*N* = 82)	Non-probiotics (*N* = 100)	*P*-value
Gender, *n* (%)			0.918
Male	56 (68.3)	69 (69.0)	
Female	26 (31.7)	31 (31.0)	
Age, years	64 (55, 70)	63 (54, 68)	0.452
Smoker, *n* (%)			0.721
Yes	38 (46.3)	49 (49.0)	
No	44 (53.7)	51 (51.0)	
Drinker, *n* (%)			0.943
Yes	25 (30.5)	30 (30.0)	
No	57 (69.5)	70 (70.0)	
Highest level of education, *n* (%)			0.081
Higher professional education	9 (11.3)	2 (2.1)	
Secondary education	43 (53.8)	55 (57.3)	
Primary education	22 (27.5)	33 (34.4)	
Illiteracy	6 (7.5)	6 (6.3)	
Unknown	2	4	
Residence, *n* (%)			0.277
City	52 (63.4)	71 (71.0)	
Rural	30 (36.6)	29 (29.0)	
Cancer type, *n* (%)			0.992
Liver cancer	38 (46.3)	48 (48.0)	
Pancreatic cancer	22 (26.8)	25 (25.0)	
Lung cancer	21 (25.6)	25 (25.0)	
Other cancers	1 (1.2)	2 (2.0)	
Tumor size, cm	3.5 (2.1, 4.6)	3.5 (2.1, 5.0)	0.972
Stage, *n* (%)			0.557
III-IV	44 (53.7)	58 (58.0)	
I-II	38 (46.3)	42 (42.0)	
Cancer therapy			**0.004**
Only surgery/radiation therapy	25 (30.5)	51 (51.0)	
First-line therapy	45 (54.9)	45 (45.0)	
Second-line and above therapy	12 (14.6)	4 (4.0)	

*Bold value means statistically significant at P < 0.05.*

### Comparison of Anxiety and Depression Levels Between Two Cohorts

According to the HAMA criteria, patients with HAMA scores ≥ 14 were considered definitely anxiety, so we regarded cancer patients with a HAMA score ≥ 14 as anxious patients, and those with a HAMA score < 14 as non-anxious patients. Our results suggested that the proportion of anxious patients in probiotics cohort (6.1%) was lower than non-probiotics cohort (13.0%), but the difference was not statistically significant (*P* = 0.121, as shown in Table 3).

**TABLE 3:** Comparison of anxiety and depression levels between probiotics and non-probiotics cohorts.

Outcome measures	Probiotics (*N* = 82)	Non-probiotics (*N* = 100)	X^2^	*P*-value
HAMA, *n* (%)			2.409	0.121
<14	77 (93.9)	87 (87.0)		
≥14	5 (6.1)	13 (13.0)		
HAMD-17, *n* (%)			1.301	0.254
≤7	57 (69.5)	77 (77.0)		
> 7	25 (30.5)	23 (23.0)		

*HAMA, Hamilton Anxiety Scale; HAMD-17, 17-item Hamilton Depression Scale; SD, Standard Deviation.*

According to the HAMD-17 criteria, patients with HAMD-17 scores > 7 were considered depression, so we divided cancer patients into non-depression group (HAMD-17 scores ≤ 7) and depression group (HAMD-17 scores > 7). The results demonstrated that the proportion of patients with depression was similar between probiotics and non-probiotics cohort (30.5 and 23.0%, respectively, *P* = 0.254, as shown in Table 3).

Logistic regression models were used to analyze whether the difference in baseline data of two cohorts would affect the final result. The results further proved that the baseline difference in cancer therapy did not affect the conclusions (*P* > 0.05, as shown in Tables 4, 5).

**TABLE 4:** Logistic regression model analysis of the effect of cancer therapy on the correlation between probiotics and anxiety in cancer patients.

Variables	Total (*N* = 182)	Non-anxiety (*N* = 164)	Anxiety (*N* = 18)	OR	95% CI	*P*-value
Probiotics	82 (45.1)	77 (47.0)	5 (27.8)	0.402	0.133-1.215	0.106
**Cancer therapy**						
Only surgery/radiation therapy	76 (41.8)	70 (42.7)	6 (33.3)	–	–	0.487
First-line therapy	90 (49.5)	79 (48.2)	11 (61.1)	1.886	0.650-5.474	0.243
Second-line and above therapy	16 (8.8)	15 (9.1)	1 (5.6)	1.137	0.120-10.745	0.911

*OR, odds ratio; CI, confidence interval.*

**TABLE 5:** Logistic regression model analysis of the effect of cancer therapy on the correlation between probiotics and depression in cancer patients.

Variables	Total (*N* = 182)	Non-depression (*N* = 134)	Depression (*N* = 48)	OR	95% CI	*P*-value
Probiotics	82 (45.1)	57 (42.5)	25 (52.1)	1.410	0.712-2.792	0.324
**Cancer therapy**						
Only surgery/radiation therapy	76 (41.8)	58 (43.2)	18 (37.5)	–	–	0.885
First-line therapy	90 (49.5)	65 (48.5)	25 (52.1)	1.169	0.573-2.385	0.668
Second-line and above therapy	16 (8.8)	11 (8.2)	5 (10.4)	1.270	0.376-4.290	0.700

*OR, odds ratio; CI, confidence interval.*

## Discussion

We have mentioned that there are significant differences in the composition of the gut microbiome of cancer patients, psychiatric patients, and healthy individuals. Compared with healthy individuals, patients with colorectal cancer have more abundant of 11 operational taxonomic units belonging to the *genera Enterococcus*, *Escherichia/Shigella*, *Klebsiella*, *Streptococcus*, and *Peptostreptococcus*, but less abundant of 5 operational taxonomic units belonging to the genus *Roseburia* and other butyrate-producing bacteria of the family *Lachnospiraceae* (22). Similarly, one study showed that the levels of *Bacteroidetes*, *Proteobacteria*, and *Actinobacteria* were significantly increased and the levels of *Firmicutes* were significantly decreased in patients with major depression disorder compared with healthy people (21). Differences in gut microbiome in different populations may lead to different effects of probiotic supplementation on anxiety and depression levels. These differences prompted us to conducted this study, and we want to explore whether probiotics are associated with anxiety and depression levels in cancer patients. Currently, there are many probiotic products that contain different strains of probiotic bacteria. Are all probiotics useful for reducing anxiety and depression symptoms? One study suggested that probiotics consisting of specific strains of *Lactobacillus* genera and *Bifidobacterium* genera might have the potential to prevent and treat depression and anxiety disorders (23). Therefore, only patients using probiotics consisting of specific strains of *Lactobacillus* genera and *Bifidobacterium* genera were included in this study. This could effectively rule out interference with the outcomes of this study by other strains that may not be effective in improving anxiety and depression.

In the current study, we found that cancer patients supplemented with probiotics did not significantly reduce the levels of anxiety and depression, which is consistent with the conclusions of some meta-analysis for non-cancer patients (17–19). However, one study reported that supplementing with probiotics improved the clinical anxiety and stress biochemical characteristics of patients undergoing laryngectomy, and taking probiotics reduced the anxiety level of patients from HAMA 19.8–10.2 (26). Although the population in this study is also cancer patients, they mainly discussed the effects of probiotics on anxiety levels in surgical patients, which is different from our research perspective.

We know that gender is one of the common risk factors for depression and anxiety, and the prevalence is higher in women (27, 28). In addition, age also affects the development of depression and anxiety disorders (29, 30). The results of a Chinese study demonstrated that age and education level were risk factors for depression, while age and gender were risk factors for anxiety disorders (31). Chemotherapy is also thought to significantly affect the gut microbiome, which may further affect the anxiety and depression levels of cancer patients (32). Besides, studies demonstrated that the gut microbiota may be associated with the development of several cancer types, such as colorectal cancer (33), gynecological cancer (34), liver cancer, lung cancer, gastric cancer, and pancreatic cancer (35–38). Additionally, the composition of the gut microbiomes differs significantly among tumor stages (39), so the differences in cancer types and tumor stages may also affect our results. Therefore, we investigated baseline characteristics of enrolled cancer patients including age, gender, education level, residence, smoking and drinking habits, type of cancer, tumor size, stage of cancer, and cancer therapy in both cohorts, so as to exclude the influence of these factors on our results. As a result, we found that patients in both cohorts were similar with respect to these factors except the cancer therapy (*P* = 0.004). However, the results of logistic regression model analysis further proved that the baseline difference in cancer therapy did not affect the conclusions.

Over the past few years, there has been growing evidence that gut microbiomes play an important role in the hypothalamic-pituitary-adrenal (HPA) axis and stress reactivity (40). An approach between them, we call it gut-brain axis. Probiotics can influence neuroplasticity-related and neurotransmitter systems through the gut–brain axis (41). An experimental study showed that a specific strain of *Bifidobacteria* could increase the levels of plasma tryptophan and alter dopamine and serotonin turnover in the brain areas associated with mental disorders (42). Goehler et al. reported that intestinal microflora could influence neural transmission in the central nucleus of the amygdala, the paraventricular hypothalamus, and the bed nucleus of the stria terminalis which were involved in the emotions processing related to anxiety and depression (43). These studies provided a theoretical basis for supplementing probiotics for the prevention and treatment of anxiety and depression. However, these theories were based on animal models. Although clinical research results had found that probiotics were effective for major depression disorder, they were not effective for anxious and healthy people. However, studies have been inconsistent as to whether probiotics are beneficial for mild to moderate depression (17–19). The results of our study demonstrated that there was no statistical difference in the proportion of cancer patients with anxiety (6.1 and 13.0%, respectively, *P* = 0.121) and depression (30.5 and 23.0%, respectively, *P* = 0.254) between probiotics and non-probiotics cohorts. Therefore, it is not recommended to supplement probiotics to reduce anxiety and depression levels in cancer patients.

Although this study shows that probiotic supplementation does not significantly reduce anxiety and depression levels in cancer patients, some limitations of this study need to be considered. First, the sample size of our study is relatively small, which may lead to bias in our results. Second, this is a retrospective cohort study that cannot directly clarify the relationship between probiotics supplementation and anxiety and depression levels in cancer patients. Third, it is well known that the symptoms of anxiety and depression are related to many factors such as environmental factors. During follow-up, it was found that none of the patients took supplements to promote mental state, but some patients took yogurt occasionally (less than 3 times a week), which might slightly affect our conclusions. Due to our design limitations, these cannot be completely eliminated. Therefore, our results need to be confirmed in a larger sample of cancer patients with better designs, such as randomized controlled trials.

## Conclusion

Our results still suggest that there is no significant correlation between probiotics and anxiety and depression levels in cancer patients. Therefore, we do not recommend supplementing probiotics for cancer patients to prevent anxiety and depression. Moreover, high-quality RCTs are also needed to further confirm the conclusions of this study.

## Data Availability Statement

The original contributions presented in the study are included in the article/supplementary material, further inquiries can be directed to the corresponding author/s.

## Ethics Statement

The studies involving human participants were reviewed and approved by the First Affiliated Hospital, Zhejiang University School of Medicine (Approval number: IIT20210103A). The patients/participants provided their written informed consent to participate in this study.

## Author Contributions

ZY and SY designed this study. MD and SL collected the data of the patients. YZ contributed to the data analysis. ZY, QZ, and SY participated in the writing and revision of the manuscript. All authors approved the final version of the article and agreed to be responsible for all aspects of the work.

## Conflict of Interest

The authors declare that the research was conducted in the absence of any commercial or financial relationships that could be construed as a potential conflict of interest.

## Publisher’s Note

All claims expressed in this article are solely those of the authors and do not necessarily represent those of their affiliated organizations, or those of the publisher, the editors and the reviewers. Any product that may be evaluated in this article, or claim that may be made by its manufacturer, is not guaranteed or endorsed by the publisher.
